# Comparing Predicted Toxicities between Hypofractionated Proton and Photon Radiotherapy of Liver Cancer Patients with Different Adaptive Schemes

**DOI:** 10.3390/cancers15184592

**Published:** 2023-09-15

**Authors:** Lena Nenoff, Atchar Sudhyadhom, Jackson Lau, Gregory C. Sharp, Harald Paganetti, Jennifer Pursley

**Affiliations:** 1 Harvard Medical School, Boston, MA 02114, USAjpursley@mgh.harvard.edu (J.P.); 2 Department of Radiation Oncology, Massachusetts General Hospital, Boston, MA 02114, USA; 3 Radiation Oncology, Brigham and Women’s Hospital, Boston, MA 02115, USA

**Keywords:** adaptive radiotherapy, NTCP, proton therapy, liver SBRT

## Abstract

**Simple Summary:**

The availability of high-quality image guidance and fully integrated online adaptation now allows for an efficient online adaptation for photon treatments. Since proton treatments can deliver a lower dose to healthy tissue, a combination of protons with online adaptation might further reduce side effects. This study compares the calculated side effects for the liver and duodenal toxicity of hypofractionated liver cancer treatments for protons and photons in an adaptive and non-adaptive setting. The results show that the differences between photons and protons are often significant, while the differences between adaptive and non-adaptive treatment schemes are not.

**Abstract:**

With the availability of MRI linacs, online adaptive intensity modulated radiotherapy (IMRT) has become a treatment option for liver cancer patients, often combined with hypofractionation. Intensity modulated proton therapy (IMPT) has the potential to reduce the dose to healthy tissue, but it is particularly sensitive to changes in the beam path and might therefore benefit from online adaptation. This study compares the normal tissue complication probabilities (NTCPs) for liver and duodenal toxicity for adaptive and non-adaptive IMRT and IMPT treatments of liver cancer patients. Adaptive and non-adaptive IMRT and IMPT plans were optimized to 50 Gy (RBE = 1.1 for IMPT) in five fractions for 10 liver cancer patients, using the original MRI linac images and physician-drawn structures. Three liver NTCP models were used to predict radiation-induced liver disease, an increase in albumin-bilirubin level, and a Child–Pugh score increase of more than 2. Additionally, three duodenal NTCP models were used to predict gastric bleeding, gastrointestinal (GI) toxicity with grades >3, and duodenal toxicity grades 2–4. NTCPs were calculated for adaptive and non-adaptive IMRT and IMPT treatments. In general, IMRT showed higher NTCP values than IMPT and the differences were often significant. However, the differences between adaptive and non-adaptive treatment schemes were not significant, indicating that the NTCP benefit of adaptive treatment regimens is expected to be smaller than the expected difference between IMRT and IMPT.

## 1. Introduction

The overall incidence of liver cancer is increasing worldwide. While resection and transplantation are currently the main treatment options, not all patients are eligible for these procedures, leaving external beam radiotherapy as a locally ablative treatment option for these patients [1]. The dose that can be delivered to the tumor is often limited by the low dose tolerance of the liver and gastrointestinal (GI) organs. Common side effects observed in patients with liver cancer treated with radiotherapy are radiation-induced liver disease, gastric bleeding, or ulcerations/perforations [2,3,4]. Reducing the dose to sensitive GI structures is critical to minimizing side effects.

Compared to photon therapy, proton therapy reduces the dose to healthy tissue and has shown promising outcomes for liver cancer patients [5,6]. However, proton dose distributions are highly sensitive to anatomical changes, and even small differences can result in underdosing the tumor or overdosing the surrounding healthy tissue [7,8]. This is particularly important if daily variations are not accounted for during treatment planning [9,10,11].

With daily high-quality 3D imaging, patient setup can be more precise and interventions such as online adaptation are possible. This can allow for setup margin reductions, which reduce the dose to the healthy liver and GI structures and may therefore allow for dose escalation and rigorous fractionation schemes such as stereotactic body radiation therapy (SBRT) [12]. Performing daily online adaptive treatments can allow for further margin reduction, as online adaptive treatments can account for daily variations in patient positioning and anatomical deformation [13,14]. Fully integrated photon treatment devices, such as MRI linacs [15,16] or Ethos [17], are now commercially available and allow for online adaptive treatments in clinical practice.

While proton therapy is expected to have advantages in organ-sparing over photon therapy for liver cancer, it may provide a greater advantage to treat patients with online adaptive photon therapy rather than non-adaptive proton therapy. The purpose of this study is to compare the calculated liver and duodenal toxicity of adaptive and non-adaptive intensity modulated proton (IMPT) and intensity modulated photon (IMRT) liver SBRT treatments.

## 2. Materials and Methods

### 2.1. Patient Data and Treatment Plans

**Figure 1:**
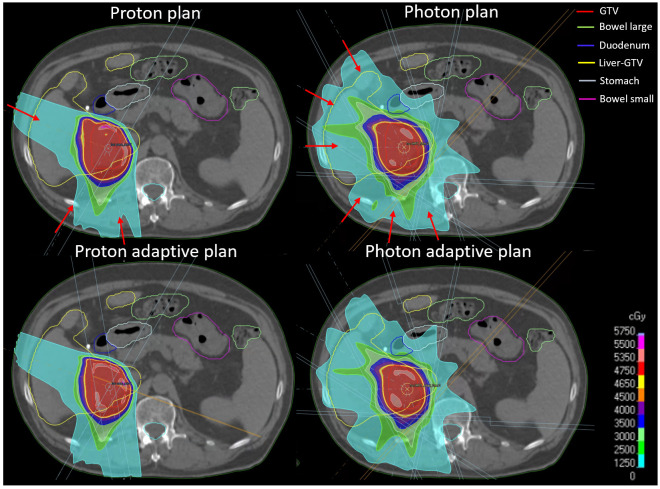
Examples of IMPT and IMRT adaptive and non-adaptive plans. Red arrows indicate beam angles.

Ten MRI linac (MRIdian, ViewRay, USA) liver cancer patients (Table 1), originally treated with 40 or 50 Gy in 5 fractions using online adaptive SBRT, were retrospectively replanned in Raystation version 10B (Raysearch, Stockholm, Sweden) for IMPT and IMRT (Figure 1) delivering 50 Gy (RBE = 1.1 for IMPT) in 5 fractions. All patients had tumors in close proximity to radiosensitive GI organs. The original MRI linac treatment plans were not used in this study, but the scans and physician-drawn contours were. The constraints used for these newly created IMRT and IMPT plans were as follows: The healthy liver (liver-GTV) mean dose had to be less than 20 Gy. The volume of duodenum, stomach, and small and large bowel receiving 35 Gy (RBE) had to be below 0.5 cc. Similar to the MR-linac treatment planning workflow, daily deformed CTs were used for dose calculation, with the planning CT deformed to the planning and daily MRI.

IMRT plans were optimized with 6 individually selected beam angles to cover the planning target volume (PTV). Non-adaptive plans were optimized to the GTV plus a 5 mm PTV margin to account for setup and calculational uncertainties and assess the potential benefit of daily adaptation. For this study, the margin for the daily adaptive IMRT plans was reduced to 2 mm after consultation with medical physicists experienced in online adaptive treatments to reflect the reduced setup uncertainties. Plans were reoptimized using a Varian TrueBeam linear accelerator (Varian, Palo Alto, CA, USA) beam model, as the ViewRay MRIdian beam model was not implemented in Raystation. IMPT plans were optimized on the GTV with three individually selected beam angles avoiding anatomical areas including air. Non-adaptive IMPT plans were optimized using a range uncertainty of 3% of the prescribed range plus an isotropic 3 mm uncertainty, including setup and calculational uncertainties. Adaptive IMPT plans were optimized with 3% range uncertainty and 1 mm isotropic uncertainty. The beam model for IMPT plans was the standard IBA dedicated nozzle beam model included in Raystation. The non-adapted workflow was simulated by recalculating the dose with the initial plan on the daily image. Daily doses were evaluated using daily structures.

### 2.2. NTCP Models

The risk of some short- and long-term toxicities can be estimated using normal tissue complication probability (NTCP) models. A widely used method for calculating toxicities is the Lyman–Kutcher–Berman (LKB) model [18,19,20,21]. In this simple model, the relationship between dose and toxicity is described by only 3 parameters: the tolerance dose for a 50% chance of complications for uniform partial organ irradiation (TD50), a parameter describing the volume effects for the organ (n), and a parameter describing the steepness of the toxicity response curve at TD50 (m).

Toxicities were calculated using two structures: healthy liver (liver-GTV) and duodenum. Three different endpoints were investigated for liver toxicity. The first was an NTCP model predicting radiation-induced liver disease (RILD) grade 3 or higher four months after the completion of radiotherapy published by Dawson et al. [22]. This model was based on 203 liver patients treated with photon therapy with a median prescribed dose of 52.8 Gy (range 24–90 Gy), out of whom 19 developed RILD. The parameters of this model were LKB parameters TD50 = 43.3, n = 1.1 and m = 0.18 with an equivalent dose of 1.5 Gy/fraction (α/β= 2 Gy). RILD is a serious side effect that is becoming increasingly rare with modern treatment modalities. Therefore, the second and third endpoints investigated are based on NTCP models developed by Pursley et al. to predict blood markers that are early endpoints for liver toxicity: the increase in albumin–bilirubin grade 1 and 2 (ALBI) (90 patients, TD50 = 32, n = 2, m = 1.5) and a Child–Pugh score increase of more than two (CP A + B) (92 patients, TD50 = 19, n = 16.67, m = 0.8) [23]. Both models are based on a mixed cohort of photon and proton radiotherapy patients with an equivalent dose of 2 Gy/fraction (α/β = 2.5 Gy), although patients received either moderate hypofractionation or SBRT. The α/β differs between the models for the same organ; to be consistent with each model, we used the respective values.

In addition, three models were used to calculate duodenal toxicity. First was a gastric bleeding model (TD50 = 180, n = 0.12, m = 0.49) based on 92 patients treated with photon radiotherapy, of which 15 patients developed gastric bleeding with an equivalent dose of 2 Gy/fraction (α/β = 2.5 Gy) [24]. Second was a grade 3 toxicity model (TD50 = 299.1, n = 0.193, m = 0.51) [25] based on 531 patients treated with photon radiotherapy (equivalent dose in 25 fractions, α/β = 4 Gy) investigating toxicities for locally advanced pancreatic cancer, gynecological cancer, and hepatocellular carcinoma. Third was a grade 2–4 duodenal toxicity model (TD50 = 24.6, n = 0.12, m = 0.23) using 73 patients with locally advanced unresectable pancreatic adenocarcinoma treated with 25 Gy photon radiotherapy in a single fraction [26]. To apply this model, an equivalent dose of 25 Gy was calculated with α/β = 4 Gy (same as in [25]).

### 2.3. NTCP Calculation

To apply each NTCP model, the dose was converted to the equivalent dose EQD [27,28,29] for the reference dose $D_{ref}$ used in each model.

$${EQD}_{D_{ref}}=D\left(\frac{fx_{dose}+\alpha /\beta }{D_{ref}+\alpha /\beta }\right)$$

The equivalent uniform dose EUD [26,30] was calculated using the EQD for each model as dose D.

$$EUD={\left(\sum_{i}D_{i}^{1/n}\frac{V_{i}}{V_{tot}}\right)}^{n}$$

This EUD was used to calculate the NTCP [23,31]:

$$NTCP=\frac{1}{2\pi }\int_{-\infty }^{t}e^{\frac{-t^{2}}{2}}dt\;\mathrm{with}\;t=\frac{\left(EUD-TD50\right)}{m\cdot TD50}$$

NTCP models assume the validity of the planned dose without considering any changes due to anatomical differences during the treatment or adjustments during an adaptive workflow. While it is common to accumulate the dose and then calculate the NTCP, adaptive plans have been optimized on structures generated with an auto contouring software and corrected by physicians (not deformed structures). Therefore, deformable accumulated doses would not be appropriate for evaluation on the structures used for planning. For consistency, we also evaluated the non-adapted plans in the same way as the adapted plans, as described below.

In order to avoid uncertainties caused by dose accumulation, the concept of ‘fraction NTCPs’ is introduced. The adaptive or non-adaptive fraction doses were scaled to the prescription dose (e.g., multiplied by the number of fractions) to calculate the fraction NTCPs. For a five-fraction treatment, this results in a set of five fraction NTCPs, which can provide an estimated mean and range of the ‘treatment NTCP’. This way, calculated ‘treatment NTCP’ is based on the delivered treatment dose rather than the planned dose and can therefore be different for different adaptive schemes of the same treatment plan, while avoiding deformation and dose accumulation uncertainties. The range of adapted and non-adapted ‘fraction-NTCPs’ was compared to the NTCPs calculated using the original dose on the planning CT. Differences between adaptive and non-adaptive IMPT and IMRT plans, dosimetric parameters, and NTCPss were tested for significance with paired *t*-tests (*p* < 0.05).

## 3. Results

**Figure 2:**
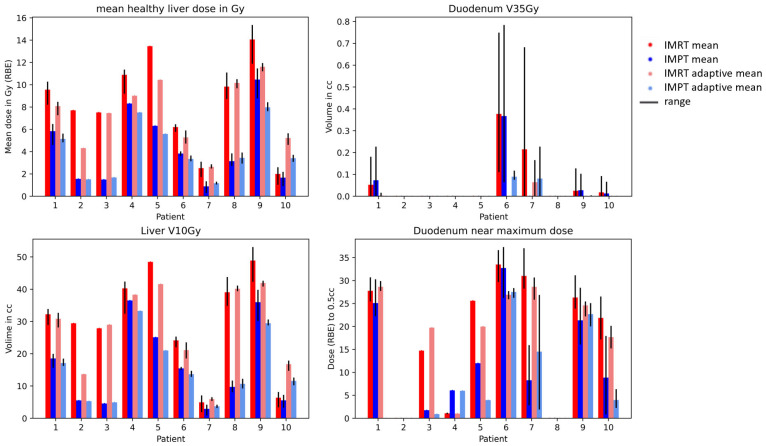
Mean healthy liver dose (liver-GTV) and duodenum V35 Gy for IMRT (red) and IMPT (blue) doses. The range over the fraction NTCPs is shown in black.

The dosimetric results for the four strategies (IMRT non-adaptive, IMRT adaptive, IMPT non-adaptive, and IMPT adaptive) are shown in Figure 2. The differences between IMRT and IMPT were significant for the mean liver dose, liver V10 Gy, and duodenum near the maximum dose in both adaptive and non-adaptive strategies, but not for the V35 Gy of the duodenum, where the high dose volume was 0 cc for most patients. The differences between non-adaptive and adaptive strategies were not significant for either dosimetric parameter, neither for IMPT nor for IMRT.

**Figure 3:**
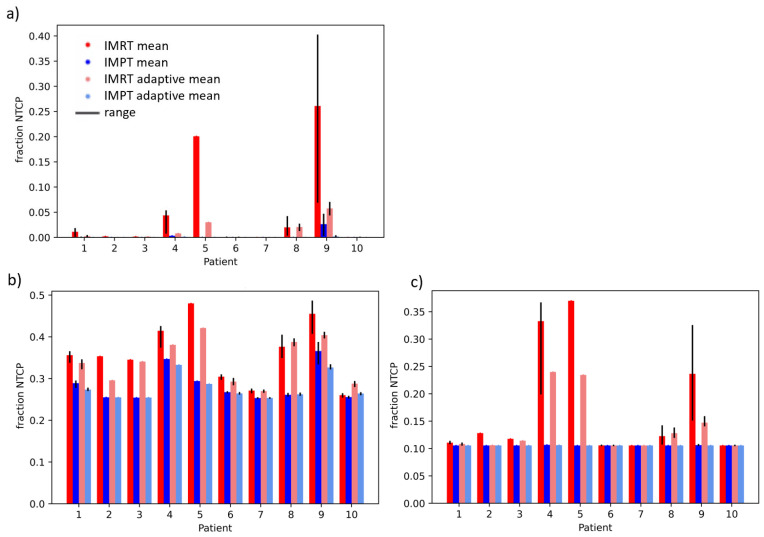
Treatment NTCPs for (**a**) RILD (Dawson et al. [22]), (**b**) ALBI, and (**c**) CP A + B (Pursley et al. [23]) for IMRT (red) and IMPT (blue) doses. The range over fraction NTCPs is shown in black.

**Figure 4:**
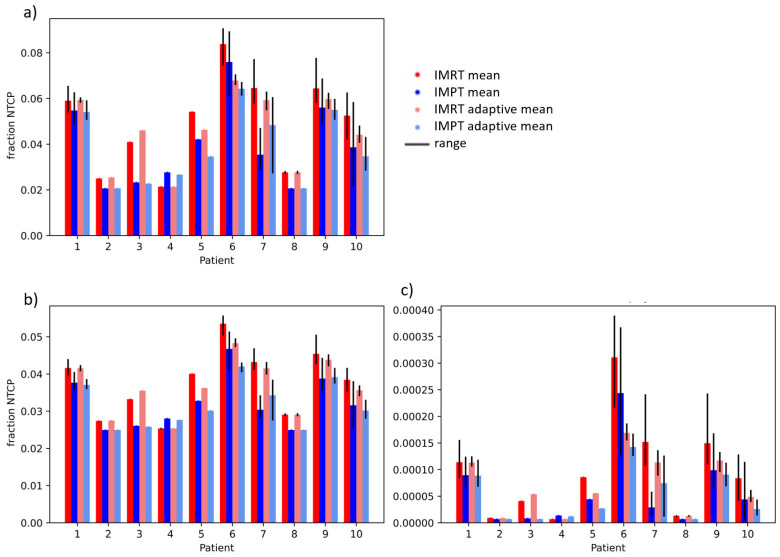
Treatment NTCPs for (**a**) gastric bleed (Pan et al. [24]), (**b**) grade 3 duodenal toxicity (Holyoake et al. [25]), and (**c**) grade 2–4 duodenal toxicity (Murphy el al. [26]) for IMRT (red) and IMPT (blue) doses. The range over fraction NTCPs is shown in black.

Figure 3 and Figure 4 show the fraction and treatment NTCPs for liver and duodenal toxicity with the different models, respectively. IMRT plans generally showed higher NTCP values than IMPT plans for the same patient. Note that, for some models (e.g., liver CP A + B or Albi), the minimum prediction (with no dose at all to the organ) is greater than 0, reflecting the high likelihood of side effects caused by the disease itself, independent of the treatment. The differences between adaptive and non-adaptive NTCPs for the same treatment modality were not significant (Table 2). On the other hand, the difference between photons and protons was significant for the Albi score and all duodenal toxicity models, but not for the Dawson RILD model and the Pursley CP A + B model, for both adaptive and non-adaptive treatments, even if the absolute differences were small.

## 4. Discussion

The differences in the estimated NTCPs between adaptive and non-adaptive treatment schemes were not significant, neither for IMRT nor for IMPT. This is surprising, especially considering the smaller margins for adaptive compared to non-adaptive treatments. The differences between the side effects of IMRT and IMPT were significant for the calculated duodenal toxicity with all NTCP models, as well as for an increase in Albi score of 2 or more as an early marker for liver toxicity. The differences in side effects between adaptation and no adaptation appear to be smaller than the differences between IMRT and IMPT.

While the high image quality of the MRI linac is crucial to confidently deliver fraction doses as high as 10 Gy with reduced margins, with the treatment being completed within one week, the resulting anatomical changes are relatively small and non-systematic in these patients (e.g., daily bowel movements but no systematic weight loss). This hypofractionation and consequently short treatment duration is likely the reason why the NTCPs did not show significant differences between the adaptive strategies. Larger and systematic anatomical changes, such as weight loss, typically occur later. The combination of MRI guidance and IMPT for liver cancer patients has been previously investigated [32]. The differences to our study include different adaptive strategies by using different margins on the planning image, not considering anatomical changes, and using different NTCP models. They confirmed a significant reduction of NTCPs between IMRT and IMPT, but they also showed a significant difference between adaptive strategies using different margins, which we did not see. This might be due to a difference in applied motion mitigation strategies for online adaptive and non-adaptive strategies in the previous study, while we assumed the same for all delivery scenarios.

The margins between adaptive and non-adaptive IMRT and IMPT were different because, in clinical practice, the reduced setup and anatomical uncertainty should be reflected in an appropriate margin reduction. Furthermore, we applied different margins for IMRT and IMPT plans because IMPT plans are optimized with robust optimization, where a uniform and a range-specific component are used, while photon plans are optimized with a uniform PTV expansion. Therefore, the incorporation of calibration and dose calculation uncertainties is included differently between the different beam modalities in clinical practice. In addition, the number of fields differed between IMRT and IMPT because an acceptable treatment plan could not be achieved with only three photon beams, but was possible for proton plans. Using the same number of beams and margins for IMRT and IMPT does not reflect clinical practice and was therefore not performed.

The mean liver dose was significantly reduced when using IMPT compared to IMRT. The maximum dose in the duodenum was not significantly changed, because it was either close to the tumor or far enough away to avoid high doses. The significant reduction in mean liver dose did, however, not translate into a significant reduction in RILD-NTCP, even though this model (Dawson et al.) is based on a near-mean dose (n = 1.1). The other liver toxicity models by Pursley et al., Albi and CP A + B, are based on n-parameters closer to minimum dose parameters (n = 2 and n = 16.67). While the Albi NTCP differences between the treatment modalities were significant, the CP A + B NTCP differences were not. A possible reason why CP A + B and RILD toxicity differences were not significant is because the minimum possible NTCP was often predicted for all investigated scenarios. For the duodenum, while none of the duodenum V35 Gy (RBE) dose differences were significant, all duodenum NTCP models, which all used close-to-maximum doses in their prediction with n < 0.2, showed a significant difference between IMRT and IMPT plans. For the liver cancer patients in this study, the duodenum V35 Gy (RBE) doses were not always constraining; in some patients, the duodenum was further away from the target. Recent trials for the stereotactic MR-guided on-table adaptive radiation therapy (SMART) of locally advanced pancreatic cancer [33] suggest advantages of daily IMRT adaptation in abdominal SBRT patients (compared to non-adaptive IMRT) if the prescription dose is high and bowel structures are directly abutting.

In this study, the dosimetric parameters used for plan optimization were different from the ones used in the toxicity evaluation. Changing the dosimetric prescription based on NTCP models is currently not clinical practice in most clinics, since the error bars of the NTCP models are not well quantified and understood. With a better refinement of NTCP models in the future, NTCP-based planning might improve the predicted patient outcome.

One method to compare doses of different treatment schemes would be to accumulate the recalculated or adapted doses with deformable image registration on the planning image and evaluate the doses with the planning structures [34]. This is not feasible in this study due to manually defined or corrected structures used for plan adaptation. For example, even if every day the target and organ doses are fulfilled in each fraction, uncertainties due to deformable dose accumulation could cause constraints to be missed when evaluated on accumulated doses. Therefore, the concepts of fraction NTCPs and treatment NTCPs are chosen in this study.

NTCP models are a useful tool for quantifying expected toxicity, but they do have several weaknesses. First, most current NTCP models are based on photon data only and consider a small number of patients and a fractionation scheme different from the one used in this study. To apply models based on different or multiple fractionation schemes, the EQD must be calculated. EQDs calculated with the linear quadratic model do have considerable uncertainties, especially for large fractionation doses [34,35,36,37]. In addition, the assumption of a constant RBE of 1.1 for proton doses is likely to result in an underestimate of the calculated NTCPs [38]. With these limitations, it might be that the current models of liver and duodenal toxicity are just not sensitive enough to detect any effects that different adaptive strategies might have on toxicity.

Recently, new model developments using machine learning and artificial intelligence have been proposed [39,40]. While machine learning models incorporate additional patient-specific information, they require large data sets and often suffer from the same weaknesses regarding patient cohort selection and different patient prescriptions/treatments in the application scenario. NTCP models including data from large cohorts of IMRT and IMPT, specific to hypofractionation (or other fractionation schemes), and incorporating patient-specific risk factors could improve the model performance. In this study, the focus was on comparing different treatment schemes for the same patient assessing dosimetric effects caused by anatomical changes during treatment and not on predicting the risk for individual patients.

A paradigm shift in NTCP models, going from planned dose to delivered dose, would likely improve their predictive power. These data can already be generated through daily imaging, auto segmentation, and automatic dose reconstruction. Although these treatment dose-based calculated NTCP models would not directly be applicable before treatment, for example for patient selection purposes [41], they might be applicable for retrospective analysis.

The lack of significant differences in NTCPs between the different adaptive strategies observed in this study does not allow for general conclusions for all liver cancer patients. The patients included in this study had short treatment times and consequently small anatomical changes between fractions. Patients with larger and/or more systematic anatomical changes during the treatment or treated with longer overall treatment times might still benefit from adaptation. Randomized clinical trails comparing different adaptive strategies and beam modalities for liver cancer patients are needed to compare observed toxicities and develop new standards for the treatment of liver cancer patients.

## 5. Conclusions

The predicted NTCPs for the liver (RILD, increase in Albi and CP A = B score) and duodenum (gastric bleeding, GI toxicity with grade >3, and grade 2–4 duodenal toxicity) were generally lower for IMPT than IMRT. While adaptive treatments have the potential to improve dose conformity, the benefit of NTCPs in a hypofractionated setting was not significant, neither for IMRT nor for IMPT. This suggests that the improvement in NTCPs using adaptive treatment workflows is expected to be smaller than the expected difference between IMRT and IMPT.

## Figures and Tables

**Table 1:** GTV and healthy liver volumes from the planning MRI for the 10 liver cancer patients.

Patient	GTV Volume in cm^3^	Liver-GTV Volume in cm^3^
1	340.05	1983.80
2	20.67	1434.67
3	92.88	1790.45
4	28.94	1165.49
5	85.56	1590.77
6	100.92	2659.70
7	20.54	1128.75
8	59.77	1061.49
9	89.78	964.46
10	26.86	1134.98

**Table 2:** *p*-values of paired *t*-tests between adaptive and non-adaptive IMRT an IMPT treatment; significant *p*-values (*p* < 0.05) are marked in bold.

Structure	NTCP Model	IMRT vs. IMPT, Non-Adaptive	IMRT vs. IMPT, Adaptive	Adaptive vs. Non-Adaptive, IMRT	Adaptive vs. Non-Adaptive, IMPT
Liver	Dawson RILD	0.103	0.078	0.121	0.302
	Pursley ALBI	**0.001**	**0.001**	0.065	0.145
	Pursely CP A + B	0.064	0.074	0.066	0.168
Duodenum	Pan gastric bleed	**0.009**	**0.010**	0.091	0.519
	Holyaoke grade 3 tox	**0.002**	**0.001**	0.098	0.433
	Murphy grade 3–4 tox	**0.011**	**0.002**	0.091	0.393

## Data Availability

The data are not publicly available due to privacy restrictions.
